# Determination of complete mitogenome sequence for Eastern Asian population of *Cheilonereis cyclurus* (Harrington, 1897) (Polychaeta: Nereididae)

**DOI:** 10.1080/23802359.2017.1372717

**Published:** 2017-09-18

**Authors:** Taeseo Park, Sang-Hwa Lee, Myung-Hwa Shin, Won Kim

**Affiliations:** a Animal Resources Division, National Institute of Biological Resources, Incheon, Korea;; b School of Biological Sciences, Seoul National University, Seoul, Korea;; c Graduate Program in Cellular Biology and Genetics, Colleage of Medicine, Chungbuk National University, Cheongju, Korea;; d National Marine Biodiversity Institute of Korea, Seocheon, Republic of Korea

**Keywords:** *Cheilonereis cyclurus*, commensal ragworm, complete mitogenome, Nereididae

## Abstract

Complete mitogenome sequence for eastern Asian population of *Cheilonereis cyclurus* (Polychaeta: Nereididae) was determined for the first time. The length of circular genome of *C. cyclurus* is 14,917 bp including 13 protein-coding genes, two rRNA genes, 22 tRNA genes, and a non-coding region of 383 bp. The gene order of *C. cyclurus* is identical to that of the following four nereidid species: *Hediste diadroma*, *Namalycastis abiuma*, *Paraleonnates uschakovi*, *Tylorrhynchus heterochaetus*. The phylogenetic position of *C. cyclurus* compared to 16 selected polychaetes was conducted and present species is closely related to the clade containing *Perinereis nuntia, P. aibuhitensis*, and *Platynereis dumerilii* with high bootstrap value.

The genus *Cheilonereis* Benham (1916) belongs to family Nereididae Blainville (1818) and has two nominal species: *C. cyclurus* (Harrington 1897) and *C. peristomialis* Benham (1916) (Bakken and Wilson 2005; Read and Fauchald 2017). They are commensal with hermit crabs. Of these, *C. cyclurus* is widely distributed in the east and west coasts of the north Pacific (Harrington 1897; Berkeley and Berkeley 1948; Imajima 1972; Paik 1977). This species is easily distinguishable among nereidids with unique characteristic of ventral peristomial flap at the oral ring. However, recent phylogenetic study using morphological characteristics revealed an ambiguous relationship with *Perinereis* species (Bakken and Wilson 2005). Also, the comparison of partial mitochondrial COI sequences revealed significant difference between east and west north Pacific populations (Figure 1(A); personal data of the author). Although, the comparison of complete mitogenome sequence may provide more valuable evolutionary information for phylogenetic studies (Chen et al. 2016), only seven nereidids are available (Boore 2001; Won et al. 2013; Kim et al. 2014; Chen et al. 2016; Kim et al. 2016; Lin et al. 2016; Park et al. 2016). The purpose of this study was to determine the complete mitogenome sequence for eastern Asian population of *C. cyclurus*.

**Figure 1.**
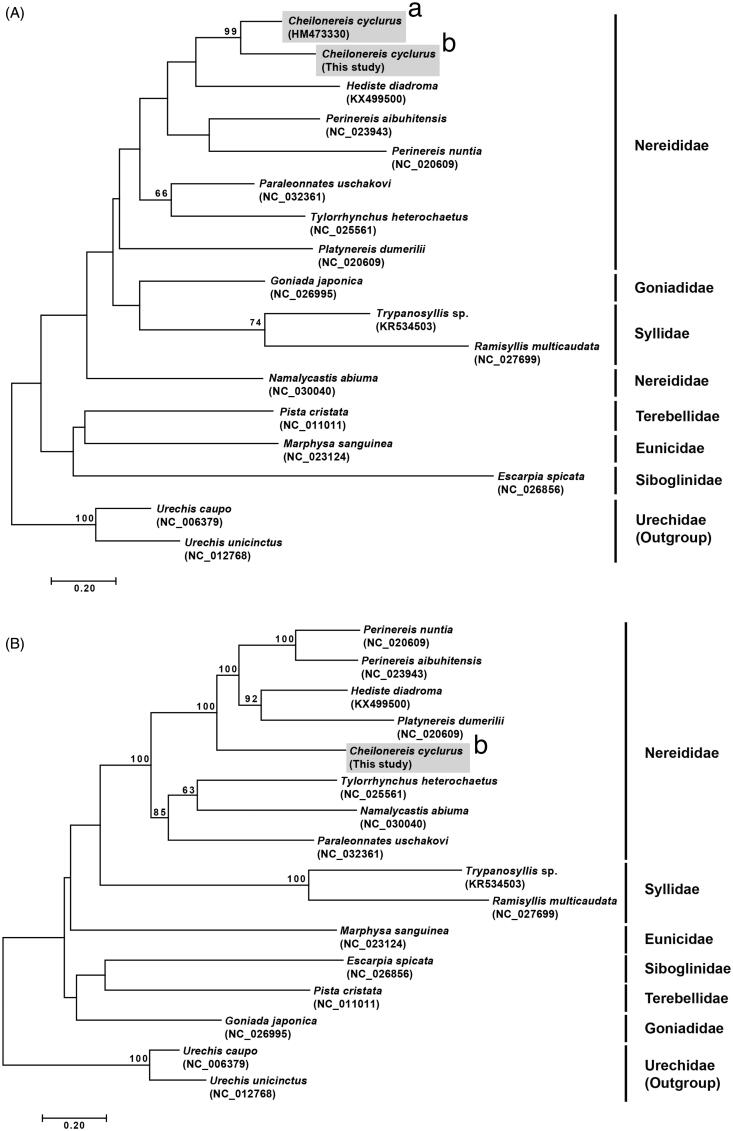
Maximum-likelihood (ML) trees constructed using MEGA 7.0 software. Bootstrap replicates were performed 1000 times. Bootstrap values above 60% were indicated on the cladogram. (A) ML tree based on 16 mitogenome sequences including *Cheilonereis cyclurus* (this study). (B) ML tree based on 17 partial mitochondrial cytochrome c oxidase subunit I (COI) including *C. cyclurus* from the Northeast and Northwest Pacific. (a) Specimen from Bamfield, British Columbia, Canada (Carr et al. 2011); (b) specimen from Goseong-gun, Gangwon-do, Korea.

The specimen was collected in the subtidal zone (about 200 m depth) of the East Sea near Goseong-gun, Gangwon-do, Korea with local fishermen’s gill net. A live specimen was used to extract pure mitochondrial genomic DNA. Total mt DNA was extracted using the DNesy Blood & Tissue Kit (Qiagen) according to the manufacturer’s protocol. mt DNA was amplified using REPLI-g mitochondrial DNA kit (Qiagen) to increase concentration for NGS analysis. A voucher specimen was housed at the National Institute of Biological Resources (NIBRIV0000787920).

Total length of the *C. cyclurus* complete mitogenome was 14,917 bp (GenBank accession no. MF538532), and encoded 37 genes (13 protein-coding genes, two rRNA genes, and 22 tRNA genes) and a non-coding region of 383 bp. According to Park et al. (2016), nereidids have two types of gene order groups depending on the patterns of following regions: tRNA-Tyr, ATP8, tRNA-Met, and tRNA-Asp (Group 1) and tRNA-Met, tRNA-Asp, ATP8, and tRNA-Tyr (Group 2). Gene order of *C. cyclurus* is identical to Group 1. The initiation codons of genes include ATT (nad1) and ATG (cox1–3, nad6, cytb, nad5, nad4L, nad2–4, atp6, atp8). Stop codons include TAA (cox2, cytb, nad2–4, nad4L, atp6) except some genes (cox1, cox3, nad1, nad5, nad6, atp8) terminated with T.

To examine the phylogenetic position of *C. cyclurus*, phylogenetic analyses were conducted using MEGA7 (Kumar et al. 2016) and reconstructed the tree in maximum-likelihood method by concatenated 13 protein coding genes from 16 selected polychaetes. As a result, *C. cyclurus* was grouped into family Nereididae and closely related to the clade containing *Perinereis nuntia*, *P. aibuhitensis*, and *Platynereis dumerilii* with high bootstrap value (Figure 1(B)).

The new mitogenome sequence determined from this study will be useful for further taxonomic and phylogenetic examination not only for eastern Asian population of *C. cyclurus* but also other nereidid species.
